# Contactless Discharge-Driven Method for Separation of Oil-Water Mixtures

**DOI:** 10.3390/mi13101652

**Published:** 2022-09-30

**Authors:** Qiang Tang, Xiaxia Cui, Zhibin Hu, Shaotian Lu, Chengjun Wang, Jau Tang

**Affiliations:** 1 School of Artificial Intelligence, Anhui University of Science and Technology, Huainan 232000, China; 2 Institute of Technological Sciences, Wuhan University, Wuhan 430072, China; 3 School of Engineering, Westlake University, Hangzhou 310024, China

**Keywords:** corona discharge, oil–water separation, needle–plate electrode

## Abstract

Oil–water separation technology has potential applications in wastewater treatment, petroleum refining and edible oil processing. As the ultimate means in oil-water treatment, electrostatic coalescence technology has been widely used in oil fields and refineries. However, the technology has many problems, such as complex processes, electrode corrosion, and the inability to treat high-water-cut crude oil emulsions. Here, we propose a contactless method of oil-water separation by corona discharge. With corona discharge of a needle–plate electrode configuration, the oil droplet diffuses to the ITO glass surface and the water droplet oscillates at the edge of the PET film. Here, such droplet behaviors are described in detail. Based on the motion behavior of the oil and water droplet, we designed an efficient oil-water separation device. After the oil-water mixture passes through the device, the oil content in the oil region can reach 99.25% with a voltage of 8 kV. In addition, the separation speed of the oil-water mixture can also be adjusted by varying the corona discharge voltage. This paper presents a simple and innovative method for oil-water separation.

## 1. Introduction

With economic growth, the output of oily wastewater in industrial production and everyday human life has increased [1,2,3]. Therefore, treating oily wastewater to obtain qualified pure oil or to produce pure water can protect the environment and bring huge economic benefits [4,5]. Gravity sedimentation is the most commonly used method for oil-water separation [6]. However, this method has a low oil-water separation speed and is only suitable for treating oil-water mixtures with large water droplet sizes. By accelerating the coalescence of tiny water droplets in the oil-water mixture into large particle-size droplets, biodegradation methods [7], heating methods [8,9], chemical methods [10], air floatation methods [11], ultrasonic methods [12,13], and some oil-water separation methods, combining physical, chemical, and biological processes, have been developed [14,15,16]. In particular, membrane separation technology is attracting increased attention for oil/water separation because of its cost-efficiency and stable performance [17,18,19]. Nevertheless, these methods still present problems, such as low separation efficiency, the production of secondary pollutants, and difficulties in separating complex oil-water mixtures during oil-water separation [20].

Compared with other oil-water separation methods, the electro-coalescence method shows unique features, such as cleaning benefits, high efficiency, and large processing capacity [21]. It mainly relies on an electric field to polarize the droplets to generate an electric field force, promote the coalescence of the droplets and accelerate sedimentation and separation [22]. Since Dr. Cottrell applied for the first crude oil electric dehydration patent in 1909, the electro-coalescence method has been continuously developed [23]. At present, it can achieve excellent performance in waste oil treatment in combination with a centrifugal field [24]. However, the electro-coalescence method is only applicable to oil-water mixtures with low water content, and the separation process is prone to water droplet chain effects, electric breakdown, electrode corrosion, and other problems, which hinder the broad application of electro-coalescence technology [25,26,27,28]. Therefore, in the face of the challenge of the high-water content of oil and optimization of the coalescence process, it is essential to urgently develop small-scale, non-contact, and widely applicable oil-water separation technology.

Many methods based on electrohydrodynamic (EHD) principles to generate, drive, and classify droplets, have been proposed, which highlight the potential for application of EHD technology for oil-water separation [29,30,31,32]. Here, we propose a contactless oil-water separation method on a half-covered plate electrode by corona discharge. Unlike droplet coalescence by electric field polarization, which has low dehydration efficiency and slow separation speed, this method has a high dehydration rate, and the voltage at the tip of the needle determines the separation speed of the oil-water mixture.

## 2. Experimental Setup

**Figure 1:**
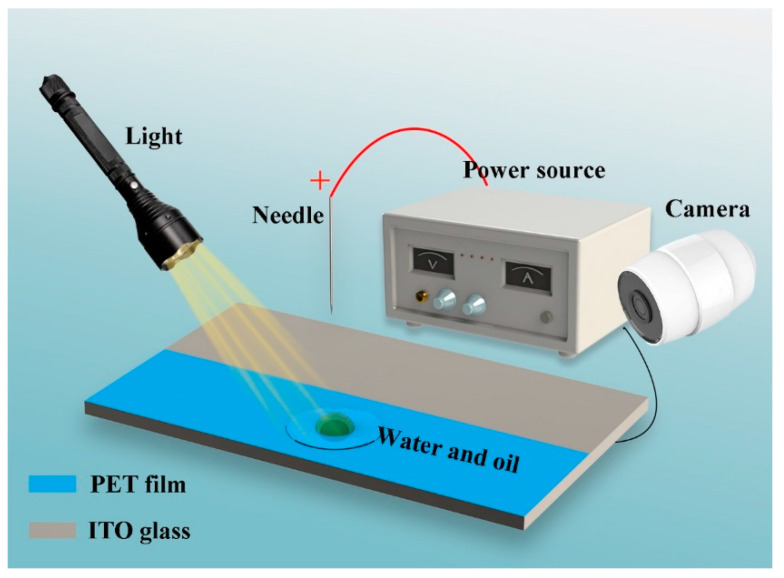
Schematic diagram of the experimental setup.

The experimental setup is shown schematically in Figure 1. A needle-plate electrode configuration was set up to generate corona discharge. The plate electrode was ITO glass (Xiangcheng & Technology, Shenzhen, China) with a resistance less than 6 Ω and size of 100 × 100 × 0.5 mm^3^. A piece of polyethylene terephthalate (PET) blue film with a surface roughness of 1.76 nm (Huajiu, Shenzhen, China) covered one side of the ITO glass with size of 100 × 50 × 0.5 mm^3^. The needle was placed vertically 40 mm above the center of the PET film and fixed with a platform. The needle was connected to the positive electrode of a high-voltage direct current (DC) power source (Dongwen Corp., Dalian, China), which can supply voltages varying from 0 kV to 30 kV. The ITO glass was connected to the negative electrode of the high-power source. The working fluid was deposited onto the PET film with a micro-syringe for each test, The experiments were conducted at ambient temperature and pressure conditions. To visualize the dynamic behavior of the droplet, a high-definition camera (Sony Corp., Tokyo, Japan) with a Laowa 60 mm lens (Laowa, Wuhan, China) was employed. A light source was located on the opposite side.

## 3. Results and Discussion

**Figure 2:**
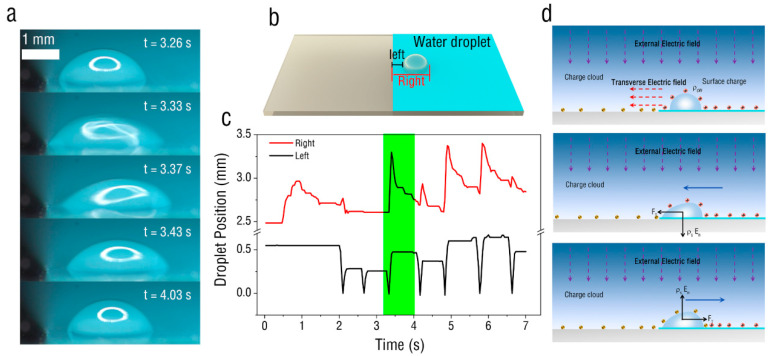
Movement of the water droplet on PET film. (**a**) Behaviors of droplet motion driven by corona discharge with voltage of 8 kV. (**b**) Schematic diagram of the experimental case. (**c**) The change in position of the left and right ends of the droplet with time. (**d**) Electrical principle of droplet movement.

At the beginning of the experiment, a deionized (DI) water droplet of volume 2 μL was placed on the PET film surface (Figure 2b). The horizontal distance between the left end of the droplet and the ITO glass was 0.55 mm. The corona voltage was kept constant at 8 kV. When the needle tip corona discharged, the position of the water droplet changed, as shown in Figure 2c. Initially, the droplet started to stretch horizontally towards the ITO glass. After 2 s, the water droplet began to oscillate at the edge of the PET film. A high-definition image for one oscillation period of the water droplet can be seen in Figure 2a (green mark in Figure 2c). First, the left end of the water droplet started to move toward the ITO glass. When it contacted the ITO glass, the water droplet started to stretch, moving to the right. Finally, the left side of the water droplet remained unchanged, and the right end gradually shrank. Therefore, we found that the water droplet on the film exhibited periodic oscillation movement accompanied by transverse stretching and shrinking driven by the corona discharge. This movement pattern of the water droplet is similar to the oscillating movement of a droplet between two electrodes in an electric field [33]; the principle is presented in Figure 2d. At the onset of corona discharge, the positive ions, generated due to the ionization process, drift towards the PET film and the deposited water droplet. Interaction of the strong electric field and the surface charges deposited on the water droplet creates an interfacial electrical pressure. Therefore, the water droplet starts to stretch. At the same time, negative charges are induced on the ITO glass, and a transverse electric field is formed at the interface to drive the water droplet to the left. Subsequently, the water droplet contacts the ITO glass and exchanges charge with it, and then moves to the right due to electrostatic repulsion. When the negative charge on the water droplet is neutralized by the positive charge on the PET film, the water droplet is again charged with the positive charge, and then the water droplet starts a new oscillation cycle.

**Figure 3:**
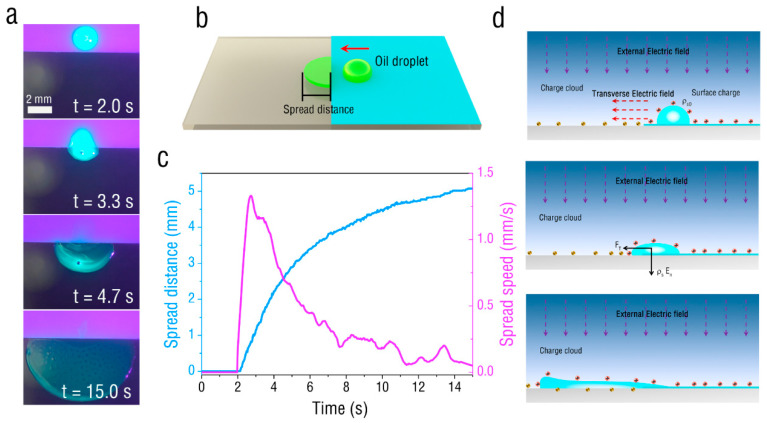
Spread of oil droplets on the ITO glass. (**a**) Spread behaviors of oil droplet driven by corona discharge with voltage of 8 kV. (**b**) Schematic diagram of the experimental case. (**c**) Variation of spread distance and spread speed of oil droplets with time. (**d**) Electrical principle of oil droplet spread movement.

Next, the water droplets were replaced by oil droplets, and the movement of oil droplets under the action of corona discharge was observed, as shown in Figure 3a. The oil droplets were of olive oil and were labeled with fluorescence. Initially, the oil droplet started to stretch horizontally towards the ITO glass. Subsequently, the oil droplets almost completely diffused to the ITO glass, and the spread area gradually increased with time. To evaluate the speed of spreading of oil droplets, the distance from the spread front end to the film boundary was taken as the spread distance, as shown in Figure 3b. In Figure 3c, the time evolution of the spread speed and spread distance of the oil droplets on ITO glass is shown. It can be seen that the spread distance of the oil droplets gradually increased with the onset of the corona discharge. The maximum spread speed of 1.32 mm/s resulted from the initial stage of diffusion (t = 2.7 s), and then the spread speed gradually decreased. The spreading principle of oil droplets is shown in Figure 3d. Under corona discharge, the oil droplets on the PET film, like the water droplets, are also subjected to interfacial electrical pressure and a transverse electric field force, driving the oil droplets to move in the ITO glass direction. Unlike water droplets, oil droplets are poor conductors of charge. After it contacts the ITO electrodes, most of the charge on the surfaces will not be rapidly conducted away, so that the oil droplet continues to be subjected to the lateral electric field force and the interface electric pressure, driving the oil droplets to diffuse to the ITO glass surface. With increase in the oil droplet spread distance, the transverse electric field force also gradually decreases.

**Figure 4:**
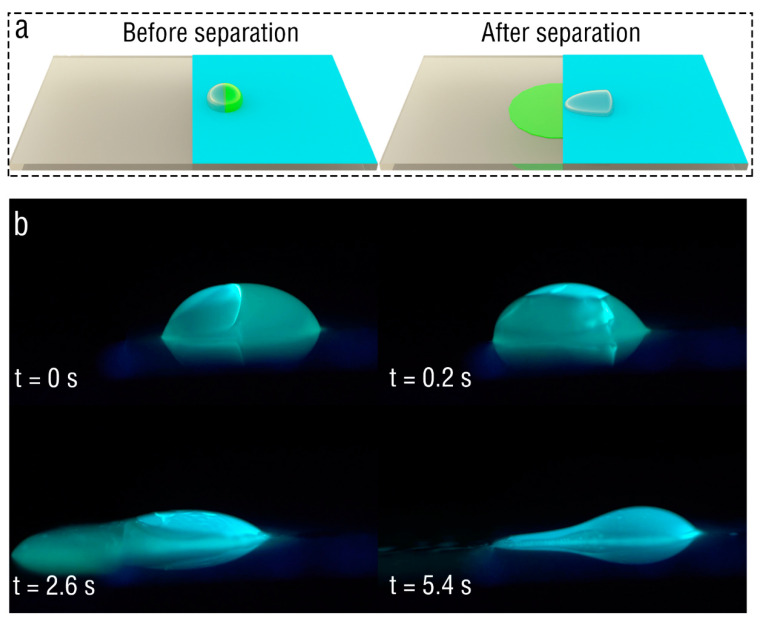
Separation of oil and water droplets. (**a**) Schematic diagram of the experimental case. (**b**) Image of separation process of the oil and water droplet under corona discharge.

The above experiments demonstrate that there are obvious differences in the areas where the oil droplets and water droplets finally occur on the plate electrode due to different conductivity. Following these, whether the contactless separation of oil and water droplets can be realized by corona discharge was tested. First, olive oil (2 μL) and DI water (2 μL) were placed on the PET film, and the oil droplet and water droplet were labeled with different fluorescence, as shown in Figure 4a. After the corona discharge was turned on, the motion behaviors of the oil and water droplets were recorded in real time using an HD camera under the irradiation of a UV lamp, as shown in the Figure 4b. The time to turn on the corona discharge was 0 s. It can be seen in the figure that, when the corona discharge acts on the oil droplet and the water droplet, the oil droplet covers the water droplet and they then move to the boundary together. When the oil droplet and water droplet contact the boundary, the oil droplet continues to diffuse to the ITO glass surface, while the water droplet stays on the PET film. It is worth noting that the surface of the water droplet is always covered with a thin oil layer, and that the oil layer gradually decreases with increase in time. The results demonstrate that the oil droplet and water droplet were separated to a great extent, confirming the effectiveness of corona discharge in separating the oil-water mixture.

**Figure 5:**
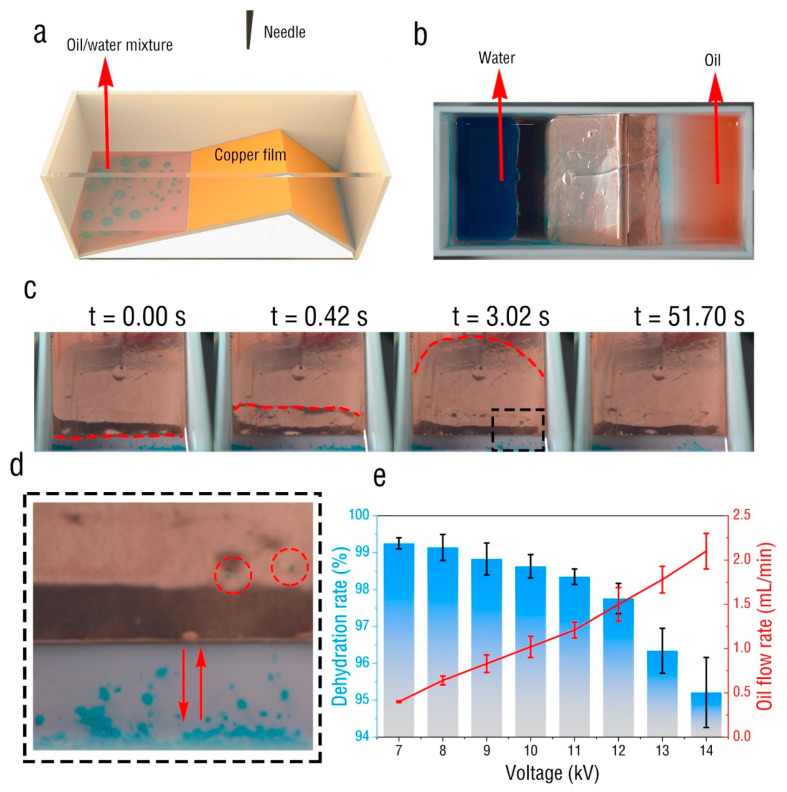
(**a**) Model of high-efficiency oil-water separation unit. (**b**) Image after oil-water separation under corona discharge. (**c**) The process of oil-water separation. (**d**) The oscillating motion of water droplet below. (**e**) The dehydration rate and the flow rate of the oil under different voltages.

Based on the corona discharge separation principle and inspired by the EHD pump [34], an efficient oil-water separation device was designed, as shown in Figure 5a. The device comprised a cuboid container of size 100 × 45 × 40 mm^3^ made by 3D-printing of photosensitive resin. The inside of the container had a slope a 16° and a length of 60 mm. The area 40 mm above the slope was covered with conductive copper film and connected to the grounding electrode. The needle tip was placed 40 mm above the center of the slope. Equal volumes of oil and water were mixed to prepare an oil/water mixture; the oil and water were dyed red and blue, respectively, for easy observation. Then the oil-water mixture was placed at the left end of the container and the liquid level of the mixture did not exceed that of the copper film. The corona voltage was set to 8 kV; when the corona discharge started, the oil gradually diffused to the copper film and finally covered the copper film, as shown in the Figure 5c. Most of the water droplets stayed at the bottom and oscillated at the boundary. However, a small portion of the liquid droplets moved on the copper film under the driving effect of the oil, as shown in the Figure 5d. Finally, the oil entered the right side of the container, while the water droplets stayed on the left side, as shown in Figure 5b. Figure 5e shows the water content in the oil and the flow rate of the oil droplets in the device under different voltages. It can be seen that, with increase in voltage, the separation speed of the oil droplets gradually increased, but the oil content in the oil region also gradually increased. The highest oil content was 99.25%, which was obtained when the corona voltage was 7 kV. This was because, when the voltage was increased, the effect on the oil droplets was increased, and many tiny water droplets were wrapped in the oil and could not be separated, so they entered the oil region.

## 4. Conclusions

In conclusion, we experimentally demonstrated a contactless oil water separation method on a half-covered plate electrode through interaction between an electric charge and droplets induced by corona discharge from a needle electrode. The oil droplet and water droplet dynamics were systematically investigated. The results showed that water droplets and oil droplets have different motion behaviors under corona discharge, with water droplets vibrating on the surface of the film, while oil droplets diffused to the grounding electrode. Based on the movement behavior of water and oil droplets, an efficient oil-water device was designed. The highest dehydration rate was 99.25% at a corona voltage of 7 kV; the separation speed was able to be adjusted by varying the corona voltage. Through use of multiple needle tips over a large area, we believe that this technology has the potential for application in several fields, such as the purification of edible oil and the collection of oil floating on the sea.

## Data Availability

Not applicable.
